# Caregiver Awareness and Knowledge of Acute Kidney Injury in Hospitalized Children

**DOI:** 10.1001/jamanetworkopen.2024.42442

**Published:** 2024-10-31

**Authors:** Michelle C. Starr, Julia Vanderkolk, Shrea Goswami, Cara L. Slagle, Danielle E. Soranno

**Affiliations:** 1 Division of Nephrology, Department of Pediatrics, Indiana University School of Medicine, Indianapolis; 2 Division of Child Health Service Research, Department of Pediatrics, Indiana University School of Medicine, Indianapolis; 3 Indiana University School of Medicine, Indianapolis; 4 Division of Neonatology, Department of Pediatrics, Indiana University School of Medicine, Indianapolis; 5 Weldon School of Bioengineering, Purdue University, West Lafayette, Indiana

## Abstract

This cross-sectional analyzes knowledge of acute kidney disease (AKI) diagnosis and associated risks among caregivers of hospitalized children.

## Introduction

Acute kidney injury (AKI) occurs commonly in pediatrics and is associated with poor outcomes.^1^ Adult patients with AKI are often unaware of their diagnoses and its impact on their future health.^2^ It is unknown whether the caregivers of children with AKI are aware of their child’s AKI diagnosis and associated risks. We sought to identify awareness and disease-specific knowledge among caregivers of children with AKI.

## Methods

This cross-sectional study was approved by the Indiana University institutional review board and followed the STROBE reporting guideline. This single-center study was performed at a tertiary pediatric referral center from May to September 2023. Pediatric participants (aged <21years) were screened for stage II or III AKI (severe AKI).^1^ Caregivers of eligible participants nearing discharge were approached; written informed consent was obtained from all caregivers. Caregivers were provided AKI information at study completion.

Caregivers self-reported demographic data and health literacy was evaluated.^3^ AKI awareness was assessed by asking, (1) “Did your child experience acute kidney injury?” and, (2) “Did your child have a problem with their kidney health?”^2^ Objective AKI knowledge was evaluated with a 9-item adaptation of the validated Kidney Knowledge Survey; scores were calculated as the percentage correct.^4^ Informational needs were assessed by asking if they felt they had enough information regarding AKI diagnosis. (eMethods in Supplement 1).

Categorical variables were summarized with frequencies and compared with Pearson χ^2^ tests. Continuous variables were summarized using medians and compared using Kruskal-Wallis tests (eMethods in Supplement 1).

## Results

Of 141 eligible patients, 96 (median [IQR] age, 3.5 [0.0-10.0] years; 48 male [50%]) were included and 65 (68%) were admitted to the intensive care unit. Of all caregivers, 72 (75%) were unaware their child had AKI and 90 (94%) were unaware of any problem with their kidneys (Figure, A). Median (IQR) AKI objective knowledge score was 60% (31%-69%). AKI recognition increased with nephrology consultation. Those admitted to neonatal intensive care unit and surgical services were less aware of AKI diagnosis (Table).

**Figure.**
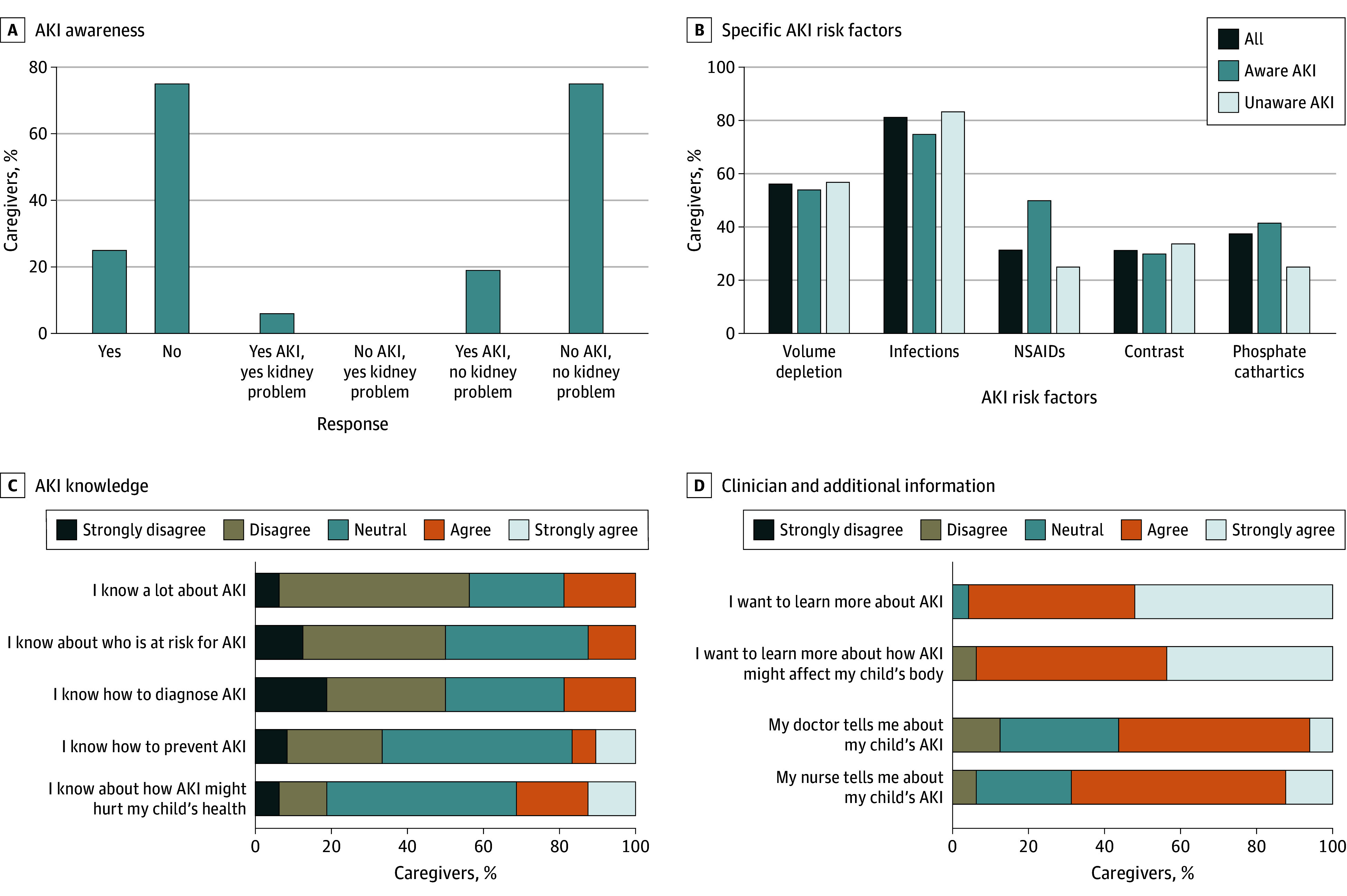
Caregiver Awareness of Acute Kidney Injury (AKI) and Kidney-Specific Knowledge A, Proportion of caregivers who reported being aware of their child having experienced AKI (yes or no); the other bars stratify this question further based on the response to the additional question, “Do you have a problem with your kidney health?” B, Identification of specific AKI risk factors. C, Caregiver responses to questions regarding AKI knowledge. D, Caregiver responses to questions regarding whether physicians or nurses met their informational needs and whether they desired to learn more about AKI in general. NSAIDs indicates nonsteroidal anti-inflammatory drugs.

**Table.** Demographic and Clinical Characteristics of Children With AKI

Characteristics	Participants, No. (%)			*P* value
	Total (N = 96)	Aware of AKI (n = 24)	Unaware of AKI (n = 72)	
Demographics				
Age, median (IQR), y	3.5 (0.0-10)	4.0 (2.0-12.5)	2.0 (0.0-11.5)	.28
Sex				
Male	48 (50)	18 (75)	30 (42)	.005
Female	48 (50)	6 (25)	42 (58)	
Race				
Black	12 (13)	0	12 (17)	NA
White	78 (81)	24 (100)	54 (75)	
Other or not disclosed^a^	6 (6)	0	6 (8)	
BRIEF literacy score, median (IQR)	18.0 (15.5-19.5)	18.5 (16.5-19.6)	18.0 (15.5-19.5)	.36
Kidney knowledge score, median (IQR), %	60 (31-69)	60 (56-65)	52 (29-71)	.55
Clinical characteristics				
Primary service				
Medical	39 (41)	13 (54)	26 (36)	<.001
Surgical	21 (22)	5 (21)	16 (22)	
Neonatology	36 (38)	6 (25)	30 (42)	
Length of stay, median (IQR), d	9 (5-57)	7 (3-8)	20 (6-70)	<.001
Peak creatinine, mean (SD), mg/dL	2.0 (1.3)	3.0 (2.0)	1.7 (0.8)	<.001
Peak stage of AKI experienced				
Stage 2	60 (63)	12 (50)	48 (66)	.14
Stage 3	36 (36)	12 (50)	24 (33)	
Required dialysis	6 (6)	4 (17)	2 (3)	.01
Nephrology consultation	42 (43)	18 (75)	24 (33)	<.001

Abbreviations: AKI, acute kidney injury; BRIEF, Behavior Rating Inventory of Executive Function; NA, not applicable.

SI conversion factor: To convert creatinine to micromoles per liter, multiply by 88.4.

^a^ Other race includes all identified racial identities other than Black or White, including participants with no information recorded, missing information, or those declining to identify a race.

Of all caregivers, 66 (69%) correctly described AKI. Most caregivers recognized dehydration (54 caregivers [56%]) and infection (78 caregivers [81%]) as AKI risk factors; however, fewer recognized risk factors such as nonsteroidal anti-inflammatory drug use (30 caregivers [31%]). Only 36 caregivers (38%) reported AKI was discussed and 88 (92%) wanted to understand more (Figure).

## Discussion

In this cohort study, we found that most caregivers of children with severe AKI were unaware of diagnosis and lacked AKI knowledge to inform post-hospital care. Many lacked understanding of AKI risk factors, with fewer than one-half aware of the potential risk of nephrotoxins. Most caregivers desired education to improve their knowledge of kidney health.

Effective health communication and knowledge is essential for transitioning care to home. Studies in pediatric patients have found that caregivers often lack understanding of their hospitalization.^5^ We suspect several reasons for low AKI awareness. First, despite adoption of standardized AKI definitions, diagnosis remains poorly recognized and clinician knowledge is variable.^1^ Second, clinicians may feel that AKI episodes are clinically insignificant, and communication is unimportant. Additionally, clinicians may be communicating ineffectively. Future studies should consider evaluating communication strategies because education is essential to post-AKI care.^6^

Our study has limitations including the use of adapted questions from adult AKI knowledge studies and single-center nature. Another limitation was timing of assessment at hospital discharge because counseling about AKI may have occurred several weeks or months prior.

In conclusion, we found that most caregivers of children with AKI were unaware of their child’s AKI diagnosis and lacked knowledge to inform post-hospital care. This knowledge is essential to reduce their risk of kidney complications.^1,6^ Closing this gap requires improved recognition, identification, and communication of kidney-specific issues with patients, caregivers, and outpatient clinicians. Studies evaluating how AKI knowledge is associated with outcomes and the role of educational interventions are needed, and future work should focus on improving communication surrounding AKI at the time of discharge.

## Notes

Supplement 1.
eMethods.

Supplement 2.
Data Sharing Statement
