# Supplementary material for: Caregiver Awareness and Knowledge of Acute Kidney Injury in Hospitalized Children
Source: JAMA Netw Open. 2024 Oct 31;7(10):e2442442. doi: 10.1001/jamanetworkopen.2024.42442 (PMC11528335; doi:10.1001/jamanetworkopen.2024.42442)
Supplement: Supplement 1. — eMethods. [file jamanetwopen-e2442442-s001.pdf]

Supplemental Online Content

Starr MC, Vanderkolk J, Goswami S, Slage CL, Sorrano DE. Caregiver awareness and knowledge of acute kidney injury in hospitalized children. *JAMA Netw. Open*. 2024;7(10):e2442442. doi:10.1001/jamanetworkopen.2024.42442

eMethods.

This supplemental material has been provided by the authors to give readers additional information about their work.

© 2024 Starr MC et al. *JAMA Network Open*.

eMethods

Parent/Guardian AKI Awareness and Knowledge Survey

Brief Health Literacy Screen (Always, Often, Sometimes, Occasionally, Never)

1. How often do you have someone help you read hospital materials?
2. How often do you have problems learning about your or your child's medical condition because of difficulty understanding written information?
3. How often do you have a problem understanding what is told to you about you or your child's medical condition?
4. How confident are you filling out medical forms by yourself?

AKI Awareness

Did you child experience acute kidney injury (AKI) while in the hospital?

No, they did not have AKI
Yes, mild AKI
Yes, moderate AKI
Yes, severe AKI

Does your child have a problem with their kidney health?

Yes
No

AKI Knowledge

1. Acute kidney injury is when:
   Your blood counts drop too low
   Your kidneys suddenly stop working well
   Your back hurts suddenly
   Your stomach hurts

2. The most common cause of AKI is:
   Taking Tylenol (acetaminophen)
   Eating too much salt
   Dehydration
   Kidney stones

3. AKI is more common if you:
   Have chronic kidney disease
   Have another medical problem
   Were born prematurely (early)
   All of the above

4. AKI is diagnosed by checking:
   Creatinine levels in your blood

© 2024 Starr MC et al. *JAMA Network Open*.

Your blood count
An x-ray
Your blood pressure

5. Severe AKI may be dangerous because it may:
Increase poisonous substances in your body
Cause you to retain fluid
Require dialysis treatments
Increase your risk of long-term kidney disease
All of the above

6. Which of the following can cause AKI?
Dehydration
Infection
Vaccines
Fleets Enemas
Giving blood
Cell phones
Motrin/Advil or Ibuprofen
Eating too much red meat

7. What may be an early symptom of AKI?
Not making as much urine
Skin turning yellow
Back pain
Headache

8. Select the ONE MEDICATION from the lis below that a person with CHRONIC kidney disease should AVOID:
Lisinopril
Tylenol/Acetaminophen
Motrin/Ibuprofen
Vitamin E
Iron pills

9. What does the kidney do? Please select one answer to each question below:
Does the kidney make urine?
Does the kidney clean blood?
Does the kidney help keep bones healthy?
Does the kidney help a person from losing hair?
Does the kidney help keep red blood cell counts normal?
Does the kidney help keep blood pressure normal?
Does the kidney help keep potassium levels in the blood normal?
Does the kidney help keep phosphorus levels in the blood normal?

AKI Provider Discussion

© 2024 Starr MC et al. *JAMA Network Open*.

Has anyone talked to you about acute kidney injury during your stay?
Yes
No, I didn't talk about acute kidney injury

AKI Perceived Knowledge (Strongly disagree, disagree, neutral, agree, strongly agree)
I know a lot about acute kidney injury
I know about who is at risk for getting AKI
I know how to diagnose AKI
I know how to prevent AKI
I know a lot about how AKI might hurt my child's health

AKI Informational Needs (Strongly disagree, disagree, neutral, agree, strongly agree)
I want to learn more about AKI in general
I want to learn more about how AKI might affect my child's body
My doctor tells me all I want to know about my child's AKI
My nurse tells me all I want to know about my child's AKI

Data Analysis

Race and ethnicity data were obtained from the electronic health record (categories included Black, White, and other [all identified racial identities other than Black or White] or not disclosed); race and ethnicity were included given existing literature demonstrating difference in caregiver awareness and communication of diagnoses between racial groups. A 2-sided $P < .05$ was considered statistically significant, and analysis was performed using Stata/SE version 18.0 (StataCorp) from February to March 2024.

© 2024 Starr MC et al. *JAMA Network Open*.
