# Supplementary material for: Caregiver Awareness and Knowledge of Acute Kidney Injury in Hospitalized Children
Source: JAMA Netw Open. 2024 Oct 31;7(10):e2442442. doi: 10.1001/jamanetworkopen.2024.42442 (PMC11528335; doi:10.1001/jamanetworkopen.2024.42442)
Supplement: Supplement 2. — Data Sharing Statement [file jamanetwopen-e2442442-s002.pdf]

Data Sharing Statement

Starr. Caregiver Awareness and Knowledge of Acute Kidney Injury in Hospitalized Children. *JAMA Netw Open*. Published October 31, 2024. doi:10.1001/jamanetworkopen.2024.42442

Data
Data available: Yes
Data types: Deidentified participant data
How to access data: Data will be made available upon request of the corresponding author.
When available: With publication

Supporting Documents
Document types: None

Additional Information
Who can access the data: Researchers whose proposed use of the data has been approved
Types of analyses: For a specified purpose
Mechanisms of data availability: after approval of a proposal and with a signed data access agreement
